# Childlessness in Twentieth-Century Spain: A Cohort Analysis for Women Born 1920–1969

**DOI:** 10.1007/s10680-018-9471-7

**Published:** 2018-03-05

**Authors:** David Reher, Miguel Requena

**Affiliations:** 1 Universidad Complutense de Madrid (UCM), Madrid, Spain; 2 Grupo de Estudios ‘Población y Sociedad’ (GEPS), Madrid, Spain; 3 Department of Sociología II, Universidad Nacional de Educación a Distancia (UNED), C/ Obispo Trejo 2, 28040 Madrid, Spain

**Keywords:** Childlessness, Fertility, Marital status, Education, Spain

## Abstract

Studies of childlessness in the twentieth century in developed countries have underscored the existence of diverging trends with higher levels among cohorts born at the beginning of the twentieth century, lower ones among the baby boom cohorts and finally higher ones for cohorts born after the Second World War. Spain also shows these basic trends, but the fit is not identical to that of other countries, with differences affecting the timing of trend changes and also the levels of childlessness observed in the final part of the period. This paper focuses on Spanish women born 1920 and 1969 and explores the factors characterizing traditional/old childlessness and how these differ from those holding more recently. Using microdata from Spanish Census of 2011, our approach makes use of logistic regression and regression-based decomposition techniques. Change over time, as measured by inter-cohort variations, reveals strikingly different patterns of behaviour characterized by a reversal of the traditional association of childlessness with marital status and educational attainment that takes place in a period of intense and pervasive social change.

## Introduction

Historical studies of childlessness point to the widespread existence of a changing trend in developed countries that start towards the end of the ninenteeth century and lasts through much of the twentieth century (Morgan 1991; Rowland 2007). See Sobotka (2016) for a detailed reconstruction of the childlessness shifts among women born between 1900 and 1972 in 28 European countries). At least two phases of this trend can be delimitated clearly: levels of childlessness decreased among the baby boom cohorts born in the early decades of the twentieth century and increased for the cohorts born after World War II. As a result of this double trend, women born in the second half of 1960s tended to exhibit very similar levels of lifelong childlessness to those women born at the beginning of the century. The same basic pattern—with only a certain delay in trend changes due to the lagged timing of its baby boom—has been documented in Spain (Requena 1997; Nicolau et al. 2010; Requena and Salazar 2014; Devolder 2015; Esteve et al. 2016) with general results indicating that levels among cohorts born in the 1960s are similar to those of cohorts born during the early part of the twentieth century.

Underlying this convergence in levels among cohorts born in distant and very different periods, and emphasizing the relationship between late marriage, fertility control and childlessness, in an influential paper published several years ago Morgan held that there was a ‘strong cultural and historical continuity in the process of producing childlessness’ (Morgan 1991: 780) during the twentieth century. While this argument is appropriate with respect to the intensity of the two peaks over the complete cycle, it is more difficult to accept in terms of its cultural and social meaning, particularly in view of the normative changes in marriage, parenthood, and childbearing associated with what has been called the ‘second demographic transition’. Doubts associated with such overarching explanations suggest the advisability of careful analysis of childlessness among cohorts born at different moments of the twentieth century, as well as a clarification of the dynamics of change between them.

In a recent paper, Hayford (2013) showed that fluctuations in childlessness among American women born in the twentieth century were the result not only of compositional variations in marital status and education, but also of long-term changes in childbearing and reproductive behaviour. In other words, increases in the proportions of never-married and highly educated women contributed to the rising overall levels of childlessness among more recent cohorts despite the fact that the proportions of childless women in these specific subpopulations decreased with respect to earlier levels. In short, the relationship between marriage, education and childlessness among cohorts born in the twentieth century appeared to change in a very profound way, relatively independent of the compositional transformation of American society.

To what extent did the reproductive behaviour of Spanish women follow a similar pattern of change during the same period? The goal of this paper is to focus on Spanish women born between 1920 and 1969 so as to analyse the factors that enable us to understand these long-term trends in childlessness. These cohorts experienced both phases of the childlessness fluctuation seen in many other developed countries. Clarifying the factors behind change over cohorts and periods will enable us to verify the extent to which historical continuities and discontinuities explain these patterns of change. In order to do so, data from the 2011 Spanish Population and Housing Census are used. The fact that Spain—along with Ireland—are the only developed nations to include information on childlessness in the most recent round of censuses increases the interest of this study because it offers detailed and up-to-date information about this important issue.

This exercise is relevant for two main reasons. Primarily, this is the first study to replicate and extend the very successful approach to childlessness implemented recently by Hayford (2013) for the USA. A major goal is to verify whether the same dynamics observed in the USA have characterized change across Spanish cohorts as well. If similar dynamics can be confirmed, the possibility of extending this kind of analysis to other developed countries could reveal the existence of a regularity relevant for a proper understanding of the secular trends in childlessness in the developed world during the twentieth century. The present paper is a first step towards this type of comparative work. Secondarily, to date most historical work on childlessness in Spain (Devolder et al. 2006; Nicolau et al. 2010) has been more focused on spatial variations than oriented to deepening our understanding of the components of long-term changes. Much of this research has tended to interpret levels of childlessness in terms of purely demographic variables at the aggregate level. We believe there is a need to delve deeper into the phenomenon by looking at individual-level data if we are to observe whether or not different forces are at work in different historical periods.

The rest of this paper is organized as follows. In Sect. 2, changes in rates of childlessness over selected cohorts in Spain are described and compared with similar estimates for other developed countries. Section 3 presents the data and methods used. Section 4 shows the descriptive results of change in composition and rates of childlessness among Spanish women, specifies a series of models for assessing the determinants of childlessness and how they change over time, and makes use of decomposition analysis, always paying particular attention to the links between childlessness and marriage and educational attainment. Section 5 concludes with a discussion of the main findings and results.

## Childlessness: The Spanish Case in Context

Historical studies of childlessness (Morgan 1991; Rowland 2007; Sobotka 2016) have shown a shifting trend in developed countries with levels increasing among cohorts born during the latter part of ninenteeth century, decreasing among the baby boom cohorts born in the second and third decades of the twentieth century and increasing again among the cohorts born after the World War II. Extending oscillations of childlessness back in time to cohorts born during the second half of ninenteeth century in several developed countries (Rowland 2007) yields further evidence of this same pattern, though estimates from further back are subject to greater uncertainty. Closer to the present, there is also evidence that, following the high levels of childlessness reached among the 1960–1964 birth cohort, there is a tendency for it to decrease in some advanced societies. While no overarching explanation for the entire change is available, and perhaps never will, the idea of long-term shifts in childlessness is an accurate description of a well-documented grand historical process.

**Fig. 1:**
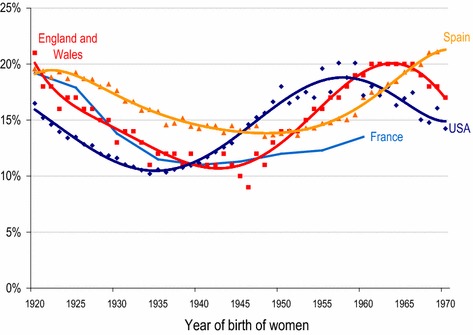
Permanent childlessness in USA, England and Wales, France, and Spain among women born 1920–1969. For USA, England and Wales, and Spain, observed data are represented by diamonds, squares and triangles, respectively; smoothed lines represent order-6 polynomial trend lines; for France, the solid line represents five-year cohort means. *Sources*: USA: 1990 USA Census for 1920–1939 cohorts, Hayford (2013) and 2012 Current Population Survey for 1940–1970 cohorts; England and Wales: Office for National Statistics, Childbearing for women born in different years, Table 2; France: Daguet (2000) and Masson (2013); Spain: 2011 Spanish Census

Figure 1 compares the rates of childlessness by cohorts born between 1920 and 1969 in the USA, England and Wales, Spain and France (five-year cohorts 1920–1964). In Spain, a very clear dual trend can be identified. Among cohorts born in the 1920s and earlier, levels of childlessness were close to 20%, decreasing to 14% for women born at the second half of the 1940s and the first half of the 1950s, and then spiking once again to 20% of women born in the late 1960s.^1^ This pattern of change is by no means peculiar of Spain.

Despite the basic similarity in patterns of change, the timing and levels of childlessness are rather different. The most visible disparity is related to the timing of basic trend changes that happen earlier in the USA (1935–1939, 1958–1962 cohorts) than in France (1940–1944), England and Wales (1945–1949, 1964–68) or Spain (1950–1954, post 1970). This timing can be explained by the fact that the demographic transition peaked a different times in these countries, as did the baby boom and the second demographic transition (van Bavel and Reher 2013; Reher and Requena 2015a). These disparities in timing, in other words, tend to be explained by larger patterns of social change taking place in different societies. Despite this, however, the basic dynamic of change appears to be similar almost everywhere in the developed world.

More difficult to explain are the differences in levels of childlessness and in the slopes of the respective curves. While initial levels of childlessness appear to have been similar in France, England and Wales, and Spain (about 20% among 1920–1924 cohorts) and higher than in the USA (about 15% among the same cohorts), cohorts born in the late 1960s diverge in important ways, with US, England and Wales oscillating around 20%, France under 15%, and Spain considerably higher. Among cohorts born 1925–1945, childlessness in Spain appears to have been systematically higher than in the other three countries and, if the graph is adjusted to control for the timing of trend changes, childlessness is always higher. With respect to the US and the British data, towards the end of the time series it is unquestionable that the earlier upward trend has come to an end and some measure of decline has begun. This is not the case of Spain where any trend change, should it take place, would happen in the future.

These fluctuations in childlessness are well known in Spain and have been aptly documented from a historical perspective (Fernández Cordón 1986; Requena 1997; Nicolau et al. 2010; Devolder 2015; Esteve et al. 2016). This literature has cited certain aggregate factors associated with these historical trends including variations in marriage practices (median age at marriage, proportions marrying), availability of spouses often linked to differentials by gender in overseas migration (sex ratios at marriageable ages and the size of the marriage market), family formation customs (average age at the first birth) and reproductive behaviour (number of children ever born). The extent to which these types of correlates are similar in the other contexts mentioned here remains to be seen and will unquestionably be a subject of future research in the field.

Making use of aggregate census results severely limits our ability to examine the underlying mechanisms behind trends in childlessness. Here micro-census data may provide a new perspective on these issues. Fortunately, information based mainly on 2011 census microdata enables us to undertake an in-depth analysis of the historical trends of childlessness in twentieth-century Spain and its dynamics that goes far beyond the more general associations normally mentioned in the existing literature. Although these new data do not provide a complete consideration of all relevant individual-level determinants of childlessness, they do enable us to undertake a systematic examination of some of the main factors behind childlessness and how they change over time. The period studied here is one of enormous societal changes and any suitable analysis should distinguish, at the very least, between structural changes in the make-up of involved cohorts (composition effects) and general changes in childbearing behaviour and attitudes towards reproduction (rate effects). The possibility of opposing effects on trends in childlessness coming from composition and rates has already been carefully observed in the USA (Hayford 2013) and cannot be discarded in Spain.

In line with the existing literature, in this paper particular attention will be paid to marital status and education, with additional controls for the region of residence, the urban/rural divide and the migratory status of women under scrutiny. Strong associations, often interpreted as causal links, between marriage and fertility—and, more specifically, between celibacy and childlessness—have been widely documented in historical and contemporary populations (Van de Walle 1972, Wunsch 1982; Coale and Watkins 1986; Wrigley 1987) and childlessness has generally been considered, at least in part, the result the proportion of women ever married. The situation may be different in post-transitional societies, with the progressive unlinking of marriage and reproduction (Pagnini and Rindfuss 1993; Muñoz-Pérez 1995; Rindfuss et al. 1980; Muñoz-Pérez and Recaño-Valverde 2011).

Secondly, the fertility of women born during the first half of the twentieth century has been shown to be negatively associated to educational attainment (Caldwell 1980; Blossfeld and De Rose 1992; Blossfeld and Jaenichen 1992; Hirschman 1994). For Spain, see Hicks and Martínez-Aguado 1987; Reher and Iriso 1989; Martín-García and Baizán 2006; Requena and Salazar 2006, 2014). Higher levels of education have been considered significant determinants of female childlessness at an individual level (Abma and Martinez 2006; Hayford 2013). A number of studies have also shown that in contemporary societies, especially among female cohorts born in the second half of the century, this negative link between female education and childlessness may be weakening or even reversing (Beaujouan et al. 2015; Tanturri et al. 2015). Recent research has found no evidence that female education results in higher proportions of childless women in Sweden (Hoem et al. 2006), Norway (Monstad et al. 2008) or Italy where education has been shown not to be a particularly good predictor of voluntary childlessness (Tanturri and Mencarini 2008).^2^ Along this line of thinking, the possibility of a shifting relationship between education and childlessness cannot be discarded in the case of Spain for more recent, post-baby boom cohorts.

Our goal is to build on the findings of relevant literature in order to track the evolution in childlessness over much of the twentieth century in Spain, explaining the main changes that have taken place and testing the extent to which the changing links between education, marriage and childlessness among more recent cohorts identified directly in the USA and indirectly in other European countries can be also found in Spain.

## Data and Method

Our analysis of childlessness in Spain is based on the 2011 Spanish Population and Housing Census microdata.^3^ For the study at hand, a broad random sample (≈ 10% of the universe) of the census data was used, made up of 4,107,465 cases (2,099,870 of whom are women). The large sample size guarantees very small sampling errors. Ten five-year cohorts of Spanish women were selected, including, at the upper age limit, women born between 1920 and 1924 and, at the younger end, women born between 1965 and 1969. These last women, who were between 42 and 46 years of age in 2011, can be considered to have virtually finished their reproductive lives at the moment the data were collected.^4^ In the census sample, the ten selected cohorts include 1,144,299 unweighted observations [weighted *N* = 1,024,157], representing a total of 11,612,962 women in the entire country.

Besides information on socio-demographic characteristics and educational attainment of residents in Spain, the 2011 census also includes information on the number of live births women have had (though not on the timing of births). These data enable us to look at childlessness for relatively recent cohorts, something that is not possible in much of the developed world where recent censuses normally do not include questions about children ever born. Although population censuses do not constitute data bases designed specifically for studying fertility, their use for this purpose is both possible and profitable. The possibility of using a single census to analyse the historical fertility trends is of particular interest (cf. David and Sanderson 1990) and has been already exploited in a number of occasions (Requena and Salazar 2006, 2014; Reher and Requena 2015a, b).

The quality of fertility data in censuses is a source of concern considering potential biases due to selective effects of mortality and migration. Problems of coverage, the tendency of informants to misreport data increasingly with age and the possibility of inaccurately reporting childlessness—as found many years ago by El-Badry (1961) and subsequently by Feeney (1995)—should be taken into account.^5^ In line with Feeney, we have checked estimates of childlessness for the same cohorts on different censuses as a rough and ready estimate of potential measurement errors. To gain precision and minimize biases, cohorts born before 1920 were discarded.^6^

An additional issue affecting our analysis is related to the fact that these data are based only on women living in households and do not include institutionalized populations. This problem is not specific to Spain and affects all modern population counts. It has the potential to lead to biases downwards in estimates of cohort childlessness because some institutionalized female populations, especially in religious institutions, tend by definition to be childless and others, in prisons or medical institutions, may also exhibit higher rates. Considering the central role of marriage for reproduction in Spain, rates of childlessness among institutional populations must have been considerably higher than rates based only on populations in households, leading to an underestimation of childlessness by as much as 1–1.5%.^7^ Despite these limitations, however, using household data provides numerous advantages when census microdata are available and so they will be used for this analysis.

In the models implemented in this paper, the following variables have been used: birth cohort (coded in ten five-year groups from 1920–1924 to 1965–1969), marital status (coded in two categories: never married and ever married), and educational attainment (coded in three categories: lower, medium and higher)^8^ are our main independent variables, with additional controls for region of residence (coded in five categories: North, North Centre, Centre, East, and South),^9^ rural/urban divide linked to city size,^10^ and migratory status (native vs immigrants).

Since the outcome of the successive decisions that produce childlessness—our dependent variable—is an intrinsically binary result, logistic regression appears to be the most appropriate statistical technique. Accordingly, we estimate several successive logistic regression models of the probability of remaining childless in order to assess the significance of the individual-level factors behind childlessness. Given the large size of the census sample, ten separate models for every five-year cohort selected will also be estimated in order to evaluate the changing associations of independent variables—in particular, marital status and educational attainment—and childlessness across the cohorts analysed. This enables us to assess the respective weights of the different predictors on the trends.

The results of a regression-based decomposition model are also included in order to quantify the contributions to change over cohorts corresponding to population composition and rates of childlessness specific to different subpopulations. The chosen technique to perform the decomposition, originally devised by Blinder (1973) and Oaxaca (1973) for continuous variables and linear regression models, was subsequently developed by Fairlie for logistic regression models with dichotomous dependent variables (Fairlie 1999, 2005). This nonlinear version has been already applied a number of times to decompose the differences between groups (Stearns et al. 2007), change over time (Van Hook et al. 2004) and change across cohorts (Hayford 2013).

The basic idea behind this technique is to decompose population-level change in the dependent categorical variable (in our case, the difference in the rate of childlessness from a given cohort to the subsequent one) into two elements: a composition component (also called characteristics effect or explained component) computed holding the models coefficients (also called rates) constant while allowing the weight of different subpopulations to vary; and a rates component (also called coefficients effect or unexplained component) computed holding the weight of independent variables (subpopulations) constant while allowing the coefficients (or prevailing rates) to vary. The interpretation of these elements is the usual counterfactual one in these kinds of decomposition: the composition component indicates how much overall rates of childlessness would have changed had coefficients for specific subpopulations not changed but their specific weight had; in turn, the rates component estimates the extent to which the overall rate of childlessness would have changed had the distribution of independent variables not varied across cohorts but the specific coefficients of childlessness for different subpopulations had.

This kind of decomposition can be implemented by means of three estimation strategies: (1) computing the rates component holding composition constant at the earlier cohort and the composition component holding coefficients constant at the later cohort; (2) computing the rates component holding composition constant at the later cohort and the composition component holding coefficients constant at the earlier cohort; and (3) computing the decomposition with coefficients from a pooled model estimated for earlier and later cohorts jointly. We have developed all three strategies, but for the sake of clarity and simplicity in the presentation of results, only the estimation from the pooled model (without including the cohorts as a control variable) will be reported in the present paper.^11^ Two tests for robustness have been applied. First, since this technique involves a one-to-one matching of cases between two cohorts with different sizes, it is necessary to draw a sample from the larger cohort. Given that results may depend on the particular sample chosen each time, it is advisable to perform a certain number of repetitions and to use average results (Jann 2006). Results reported here correspond to 1000 repetitions, although coefficients reached stability with fewer repetitions. Second, since the contributions from independent variables can be sensitive to the ordering of variables, these have been ordered randomly in each replication for the purpose of obtaining approximate results across all possible orderings (Fairlie 2005).

## Empirical Findings

### Old and New Forms of Childlessness

Table 1 shows descriptive results for all birth cohorts between 1920–194 and 1965–1969 relative to the distribution of selected covariates and the childlessness rates of different subpopulations. The upper panel of the table reports the changes of the female population of Spain over the selected cohorts with respect to their marital status, educational attainment, rural–urban divide, region of residence and migratory status. Considering the potential impact of marital status and education for inter-cohort change in childlessness, two observations are warranted. First, marital status changed over cohorts, with declining proportions of never married until women born in 1935–1939, with more recent cohorts experiencing a significant and lasting increase in proportions of never married. Second, across the selected cohorts, a dramatic structural change in levels of education of Spanish women was also taking place. Among earlier cohorts, about eight out of ten women never went beyond primary level, while in more recent cohorts born in the second half of sixties only 12% of women did not pass primary education. By contrast, whereas a tiny portion (3%) of women born 1920–1924 ended their education with a university degree, 26% of those born 1960–1969 become college graduates. This revolution in education, still only partially completed for women born 1965–1969, started rather late in comparative terms but ended up becoming one of the hallmarks of modernization in Spain with levels today similar to or even above those of the most advanced European nations.

**Table 1:** Childlessness, marital status, education, size of municipality, region and migratory status of women in Spain born 1920–1969. *Source*: 2011 Spanish Census. Data weighted by sample weights

*N*	Year of birth									
	1920–24	1925–29	1930–34	1935–39	1940–44	1945–49	1950–54	1955–59	1960–64	1965–69
	29,818	56,629	84,358	82,097	95,164	110,122	115,105	135,310	153,043	162,510
**% of all women**										
Marital status										
Ever married	90.8	91.4	92.4	93.3	93.0	92.6	91.6	89.9	87.1	83.3
Never married	9.2	8.6	7.6	6.7	7.0	7.4	8.4	10.1	12.9	16.7
Education										
Lower	81.1	80.7	77.4	71.3	58.4	45.9	32.7	22.3	16.3	12.3
Medium	15.8	15.9	18.8	24.0	34.4	44.1	53.2	58.7	61.9	61.9
Higher	3.1	3.4	3.8	4.6	7.2	10.0	14.1	19.1	21.8	25.9
Rural–urban divide										
Rural	10.4	10.4	10.1	8.9	7.4	6.4	5.8	5.5	5.5	5.0
Small cities	33.1	33.8	34.4	34.9	34.8	34.4	34.4	35.3	36.4	36.9
Intermediate	27.6	28.1	28.3	29.3	30.7	32.6	33.5	33.1	32.1	32.5
Big cities	28.9	27.7	27.2	26.9	27.0	26.7	26.3	26.1	26.0	25.6
Region										
North	19.9	18.7	18.5	17.4	16.5	16.6	16.5	15.8	14.6	14.0
North Centre	14.2	13.7	13.0	11.9	11.2	10.6	10.5	10.7	10.5	9.9
Centre	17.2	17.0	16.6	16.7	17.2	17.5	17.6	18.0	18.3	18.8
East	30.3	29.9	29.9	30.4	31.7	32.2	32.0	31.4	31.2	31.7
South	18.3	20.6	22.0	23.6	23.4	23.0	23.3	24.1	25.4	25.6
Migratory status										
Native	97.7	97.5	97.2	95.6	94.0	93.1	91.9	90.4	87.4	84.1
Migrant	2.3	2.5	2.8	4.4	6.0	6.9	8.1	9.6	12.6	15.9
**% of childless women**										
All women	19.1	18.5	16.5	14.9	14.3	14.0	14.0	15.0	17.3	20.1
Marital status										
Ever married	11.9	11.7	10.5	9.7	8.8	8.3	7.8	8.1	9.0	10.3
Never married	89.5	90.7	89.4	88.4	87.3	85.0	80.9	77.3	73.2	69.1
Education										
Lower	16.6	16.2	14.4	12.7	11.6	11.6	11.4	12.1	14.2	17.2
Medium	27.8	26.0	22.0	18.7	16.6	14.4	13.1	13.3	15.2	17.7
Higher	40.6	37.9	32.7	29.3	25.1	23.1	23.2	23.8	25.7	27.3
Rural–urban divide										
Rural	19.6	18.1	16.6	14.9	14.2	14.1	14.2	15.1	17.3	21.1
Small cities	18.5	17.4	15.3	13.8	13.2	12.4	12.0	12.3	14.0	16.8
Intermediate	18.6	18.4	16.1	14.7	13.8	13.4	13.2	14.4	17.2	19.9
Big cities	20.1	20.0	18.3	16.6	16.4	16.7	17.6	19.4	22.0	24.9
Region										
North	19.8	19.0	16.1	14.1	13.4	13.4	13.2	16.3	19.8	23.3
North Centre	19.6	18.9	18.5	16.4	15.4	14.6	15.4	16.4	18.9	23.2
Centre	19.4	18.5	17.3	16.3	15.4	15.8	16.5	17.0	19.1	21.7
East	17.9	17.4	15.4	14.2	13.4	13.1	12.6	13.9	16.5	19.1
South	19.4	19.3	16.5	14.8	14.7	14.0	14.0	13.5	14.9	17.2
Migratory status										
Native	19.0	18.3	16.4	14.5	13.9	13.5	13.5	14.7	17.3	20.6
Migrant	22.7	24.2	21.5	23.4	20.3	20.5	19.3	17.9	17.2	17.4

The lower panel of Table 1 presents the rates of childlessness among the different subpopulations. It is clear that the importance of childlessness among never-married women decreased throughout the period studied, as opposed to ever-married women where childlessness first decreased and then increased. In this way, three distinct patterns can be seen: (a) cohorts 1920–1944 where marriage is on the rise and childlessness is decreasing for everyone; (b) cohorts 1945–1969 where the importance of marriage is in decline and childlessness is increasing (8.3% → 10.3%) among ever-married while diminishing sharply (85.0 → 69.1%) among never-married women. (c) There is also a strong positive association between childlessness and education, with more educated women having higher levels of childlessness. This relationship also appears to start changing with women born after 1945: levels of childlessness of women with lower education increases substantially, while increases in childlessness among medium and highly educated women were relative smaller. As a result, for women born 1965–1969 differences in childlessness among those with lower and medium educational attainment practically disappeared and divergences from female college graduates become much smaller. In other words, in Spain the educational gradient in childlessness reduced considerably across observed cohorts.

Given that the main purpose of this study is to analyse the transition from traditional to modern forms of childlessness, a straightforward comparison between the 1920–1924 and 1965–1969 cohorts is helpful. As indicated above, the women of both cohorts exhibited similar levels of childlessness: 19.1 and 20.1%, respectively. This shared prevalence was the result of very different social and demographic forces. Important disparities in both composition and rates make these two cohorts very different.

Even though the women of the 1920–1924 cohort were young in a largely pre- or early-industrial context, they participated fully in the demographic transition. Their reproductive behaviour took place prior to baby boom and was driven by the cohorts born in 1930s. Their childlessness occurred in a context of relatively high fertility (TCFR = 2.46) and nuptiality (90.8% of ever-married women) and very low educational attainment (81.1% of these women only had primary education or less). Typical of most developed countries at this time, childlessness was positively associated with the incidence of spinsterhood and educational attainment. Yet if we look at the educational make-up of the female population born 1920–1924, it is clear that a substantial part of the number of childless women, 70.6% (= 81.1*16.6/19.1), were to be found among women of lower educational levels, and hence women from lower social strata, whereas more highly educated women of these cohorts only account for only 6.6% (= 3.1*40.6/19.1) of all childless women. In view of all these data, during this earlier period childlessness can be understood to a large extent in terms of its association with material deprivation (poverty, health problems, poor nutrition) characteristic of disadvantaged social segments with low levels of education. Even among the 90.8% of women from this cohort who married, 11.9% remained childless, a proportion slightly higher than the levels of involuntary childlessness estimated generally for pre-transitional populations.^12^

In sharp contrast to the 1920–1924 cohort, the 1965–1969 one represents an entirely new form of childlessness. Born in the second half of 1960s, these women spent their youth and most of their reproductive lives living in a modern society. With very low cohort fertility (1.54), they accurately represent the new Spanish baby bust generations, characterized by ample use of contraception and high levels of childlessness. Among these women, childlessness happened in a context of low and declining nuptiality (16.7% of them never married) and very high female educational attainment (88% achieved medium or higher levels of education), and was strongly linked to the growing numbers of women never-married. As a result, the contribution of never-married women to overall childlessness for this cohort is historically unprecedented in Spain (57.5% of all childless women never married). While the incidence of childlessness continued to be higher among more highly educated women of this cohort, differences were much smaller than before. Yet due to their increasing importance in the total female population, these advantaged strata account for 35% (= 25.9*27.3/20.1) of total number of childlessness women. By contrast, the part of all childless women corresponding to lower educated women was only 10% (= 12.3*17.2/20.1).

### Multivariate Analysis of Inter-Cohort Change

In order to go beyond these descriptive results, Table 2 presents the estimates of four successive logistic regression models for the probability of not having children among the selected cohorts. The fist model includes only ten dummy variables for the respective birth cohorts. This unconditional model basically depicts the same pattern of change of Fig. 1 and Table 1, with the 1950–1954 cohort representing the point of inflexion that marks the swing from a decreasing trend in childlessness to an increasing one. The second model adds marital status to the previous one. The association between celibacy and childlessness is, as expected, exceedingly strong (*b* = 3.599; OR = 36.56 for never-married women). When marital status is taken into account, the size of coefficients associated to cohorts born before 1945 is slightly smaller than in the earlier model, but describes the same downward trend; the coefficients for the four five-year cohorts born between 1950 and 1969, on the other hand, show almost no trend for these women, suggesting that any growth in childlessness across these cohorts must be the result of increases in the proportions of never-married women. The third model adds the educational attainment of these women. The positive relation between education and childlessness is, again as expected, very clear. In this third model coefficients for earlier cohorts (1920–1939) depict the same trend as shown in the two models not including education; but, interestingly, after the 1940–1944 cohort the coefficients for the cohorts are smaller than in the previous models and the point of inflexion of the trend moves now to the 1955–1959 cohort, with virtually no inter-cohort change after it. This points to possible composition effects of education on growth in childlessness among these more recent cohorts. Finally, the fourth model including further controls for rural–urban divide, region and migratory status is nearly identical to the third one in terms of the key variables. The rural–urban gap seems to point to a curvilinear relationship with higher levels of childlessness in relatively small populations and in big cities; geographical differences in childlessness are generally unimportant, indicating a slightly larger prevalence in the centre and east of the country; and, lastly, the coefficient for migratory status is not statistically significant.

**Table 2:** Logistic regression for probability of childlessness. Source: 2011 Spanish Census. Data weighted by sample weights. *Source*: 2011 Spanish Census. Data weighted by sample weights

	Model 1		Model 2		Model 3		Model 4	
	*b*	SE	*b*	SE	*b*	SE	*b*	SE
Intercept	− 1.445	0.015***	− 1.946	0.017***	− 1.997	0.017***	− 1.852	0.025***
Birth cohort								
1920–24 (omitted)								
1925–29	− 0.039	0.018*	− 0.016	0.021	− 0.018	0.021	− 0.017	0.021
1930–34	− 0.176	0.017***	− 0.132	0.020***	− 0.144	0.020***	− 0.141	0.020***
1935–39	− 0.295	0.018***	− 0.226	0.021***	− 0.256	0.021***	− 0.250	0.021***
1940–44	− 0.346	0.017***	− 0.321	0.020***	− 0.390	0.021***	− 0.380	0.021***
1945–49	− 0.371	0.017***	− 0.387	0.020***	− 0.494	0.020***	− 0.481	0.020***
1950–54	− 0.371	0.017***	− 0.466	0.020***	− 0.616	0.021***	− 0.601	0.021***
1955–59	− 0.288	0.017***	− 0.476	0.020***	− 0.666	0.020***	− 0.649	0.020***
1960–64	− 0.120	0.016***	− 0.436	0.019***	− 0.644	0.020***	− 0.627	0.020***
1965–69	0.064	0.016***	− 0.419	0.019***	− 0.648	0.020***	− 0.628	0.020***
Marital status								
Ever married (omitted)								
Never married			3.599	0.008***	3.561	0.009***	3.554	0.009***
Education								
Lower (omitted)								
Medium					0.205	0.008***	0.197	0.008***
Higher					0.583	0.011***	0.572	0.011***
Rural–urban divide								
Rural (omitted)								
Small cities							− 0.139	0.014***
Intermediate							− 0.134	0.014***
Big cities							− 0.057	0.014***
Region								
North (omitted)								
North Centre							0.052	0.013**
Centre							− 0.045	0.011**
East							− 0.050	0.010***
South							− 0.032	0.011
Migratory status								
Migrant (omitted)								
Native							− 0.023	0.012
− 2 log-likelihood	906,947.16		674,551.76		671,526.43		671,223.97	

*p < .05; **p < .01; ***p < .001

**Fig. 2:**
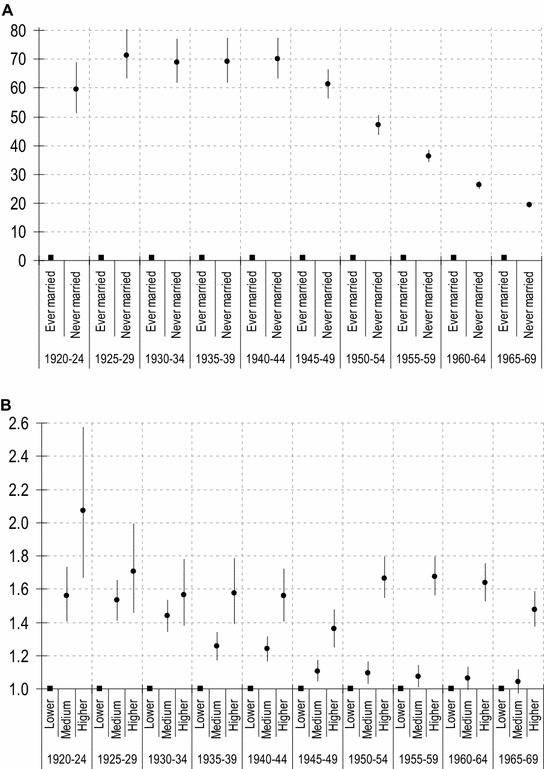
Adjusted odds ratios for childlessness by marital status and educational attainment and birth cohort (with confidence intervals). Odds ratios deduced from models in Table 3. *Sources*: 2011 Spanish Census

In order to show how our key constraints vary over cohorts, ten logistic regression models have been specified to estimate the probability of childlessness for the ten five-year cohorts born between 1920–1924 and 1965–1969 (Table 3). These ten models^13^ allow us to visualize the impact of the key predictors of childlessness net of compositional changes for each selected cohort. Although the models include the same covariates for every cohort as in the full model, here the main goal is to focus on marital status and education (Fig. 2).^14^

**Table 3:** Logistic regression for probability of childlessness. Ten selected cohorts *Source*: 2011 Spanish Census. Data weighted by sample weights

	1920–24	1925–29	1930–34	1935–39	1940–44	1945–49	1950–54	1955–59	1960–64	1965–69
	*b*	*b*	*b*	*b*	*b*	*b*	*b*	*b*	*b*	*b*
Intercept	− 2.041***	− 1.722***	− 1.950***	− 1.755***	− 1.948***	− 1.836***	− 2.215***	− 2.273***	− 2.365***	− 2.343***
Marital status										
Ever married (omitted)										
Never married	4.085***	4.267***	4.235***	4.237***	4.250***	4.114***	3.852***	3.595***	3.271***	2.956***
Education										
Lower (omitted)										
Medium	0.446***	0.426***	0.363***	0.227***	0.214***	0.102***	0.092***	0.070**	0.061*	0.039
Higher	0.728***	0.533***	0.449***	0.454***	0.443***	0.307***	0.510***	0.517***	0.493***	0.390***
Rural–urban divide										
Rural (omitted)										
Small cities	− 0.110*	− 0.072	− 0.084*	− 0.118**	− 0.062	− 0.168***	− 0.147**	− 0.190***	− 0.200***	− 0.212***
Intermediate	− 0.093	− 0.093*	− 0.123**	− 0.099*	− 0.117*	− 0.188***	− 0.213***	− 0.190***	− 0.103**	− 0.131***
Big cities	− 0.133*	− 0.105*	− 0.116*	− 0.152**	− 0.136**	− 0.173***	− 0.121*	− 0.026	0.028	0.036
Region										
North (omitted)										
North Centre	0.108	0.097*	0.172***	0.204***	0.120**	− 0.021	0.089*	− 0.072*	− 0.047	0.041
Centre	0.058	0.105*	0.126*	0.157***	0.049	− 0.005	0.087*	− 0.142**	− 0.195***	− 0.203***
East	0.040	0.095*	0.088	0.159***	0.057	− 0.006	− 0.010	− 0.170	− 0.182***	− 0.192***
South	0.124*	0.207***	0.212***	0.179***	0.140***	0.054	0.122***	− 0.191**	− 0.271***	− 0.315***
Migratory status										
Migrant (omitted)										
Native	− 0.017	− 0.432***	− 0.257***	− 0.627***	− 0.525***	− 0.534***	− 0.302***	− 0.046	0.186***	0.378***
− 2 log-likelihood	21,495.9	40,032.6	56,480.2	52,269.2	57,263.6	64,844.8	66,967.1	82,170.1	102,845.2	121,685.8
*N*	29,818	56,629	84,358	82,097	95,164	110,122	115,105	135,310	153,043	162,510

*p < .05; **p < .01; ***p < .001

Regarding marital status, among cohorts born 1920–1924 the odds were almost 60:1 (*b* = 4.08) that never-married women would remain childless in comparison to ever-married women, increasing up to 69:1 (*b* = 4.250) for women born 1940–44. From then on, there are systematic decreases in the odds of never-married women being childless reaching a low of 19:1 (*b* = 2.956) for those born 1965–9 (see Fig. 2, panel *A*). These results fully confirm the descriptive results: as appears to have happened in other developed countries, the de-linking of marriage and childbearing for Spanish cohorts born in the sixties is a result of both an increase in childlessness among married women and a decrease among never-married women (clearly visible in Table 1, lower panel).^15^ Although the association between celibacy and childlessness was always strong in Spain, its importance dwindles dramatically among the post-baby boom cohorts, and in particular among women born in the sixties. This trend is a fitting testimony of the growing relevance of different attitudes towards marriage and reproduction among these cohorts. Women born in 1960s made a significant departure from the traditional pattern of a rigid association between marriage and childbearing experienced by earlier cohorts. Since they did not have to face the normative cultural pressures that confined childbearing to the role of spouse, they began to dissociate marriage and reproduction as never before.

The change of behaviour among women of different educational levels also warrants our attention. As shown in the third and fourth models in Table 2, Spanish women born 1920–1969 exhibited a strong positive association between childlessness and education: the more educated the woman, the higher the level of childlessness. Of considerable interest is, however, the fact that this relationship appears to have changed in a relevant way across cohorts. Three patterns can be observed. (1) Among women born 1920–1934, differences in childlessness associated with middle and higher educational attainment hardly exist (see the overlapping confidence intervals of odds ratios for both levels in Fig. 2, panel *B*). In terms of overall childlessness, it was the difference separating women with lower education levels from the rest that mattered. (2) For female cohorts born 1935–1949 the differences in childlessness among the three educational levels were quite proportional. (3) Among women born after 1950 it was the distance between higher and medium/lower education what made the (only statistically significant) difference in childlessness. In fact, this process of reduction in educational disparities starts earlier, with differences between lower and medium visible from the 1930–34 cohort, followed by a reduction in differences between higher and the rest starting with the 1960–64 birth cohort. It is unclear whether this last trend in higher education will continue past 1970, but this may well be the case. The ultimate result is that the effect of education on childlessness diminishes substantially over time as the overall educational gradient declines.

### A Decomposition of Change in Childlessness

Descriptive results and regression models shown above suggest that both compositional (characteristics) and rate (coefficients) effects are relevant for explaining both the increase and decrease in the childlessness of Spanish women born 1920–1969. In order to quantify the relative contribution of each to change, a decomposition analysis based on the logistic regression models presented earlier has been implemented with the technique explained in Sect. 3. Since the trend change in childlessness is located in the 1950–1954 cohort, two inter-cohort intervals are selected for decomposition: the first one, when childlessness decreased 5.1%, includes 1920–1954 birth cohorts; the second one, when childlessness increased by 6.1%, is based on the 1950–1969 birth cohorts. Table 4 presents the results of this decomposition, specifying the respective contributions of each variable to change in both intervals.

**Table 4:** Nonlinear decompositions of changes across selected cohort in childlessness rates. *Source*: 2011 Spanish Census. Data weighted by sample weights

	Composition effects	Rate effects	Total effect
1920–1924 to 1950–1954			
Total change	− 0.004**	− 0.047***	− 0.051***
Intercept		− 0.016	
Marital status (omitted = ever married)			
Never married	− 0.007*	− 0.002**	− 0.009
Education (omitted = lower)			
Medium	− 0.005**	0.000	− 0.005
Higher	0.008	0.001**	0.010
Rural–urban divide (omitted = rural)			
Small city	− 0.001	− 0.001	− 0.002
Intermediate	− 0.003	− 0.003	− 0.006
Big city	0.001	0.000	0.001
Region (omitted = North)			
North Centre	− 0.001	0.000	− 0.001
Centre	0.000	0.000	0.001
East	0.000	− 0.001	− 0.002
South	0.001	0.000	0.001
Migratory status (omitted = immigrant)			
Native	0.003	− 0.025	− 0.022
1950–1954 to 1965–1969			
Total change	0.056***	0.005***	0.061***
Intercept		− 0.004	
Marital status (omitted = ever married)			
Never married	0.048***	− 0.003***	0.045
Education (omitted = lower)			
Medium	0.002***	− 0.001	0.000
Higher	0.010***	− 0.001*	0.009
Rural–urban divide (omitted = rural)			
Small city	− 0.001***	− 0.001	− 0.002
Intermediate	0.000***	0.001	0.001
Big city	0.000	0.001**	0.001
Region (omitted = North)			
North Centre	0.000*	0.000	0.000
Centre	0.000**	− 0.002***	− 0.002
East	0.000	− 0.002**	− 0.002
South	− 0.001***	− 0.004***	− 0.004
Migratory status (omitted = immigrant)			
Native	− 0.003***	0.021***	0.018

Coefficients from the pooled model estimated for earlier and later cohorts jointly

*p < .05; **p < .01; ***p < .001

This decomposition points to the existence of two very distinct patterns of change. Among cohorts born 1920–1954, the reduction in childlessness was dominated by rate effects (− 0.047 in the pooled model; 92% of the total change) that clearly outweighed compositional effects (− 0.004). The importance of the rates component reveals general societal changes fostering childbearing and lowering the general level of childlessness. It was no coincidence that part of this inter-cohort interval corresponds to the baby boom period. All subpopulations tended to participate in this broad trend, with the contributions to the decline in childlessness attributable above all to the behaviour (rates) of women who never married and had lower educational attainment. Changes in the characteristics of the population also tended to depress childlessness but were much less important (8% of the total change) than changes in rates. Although in the aggregate composition effects are rather smaller than rate effects, the decreasing proportion of never-married women (the marriage boom of the time) contributed to a reduction in childlessness (− 0.007, − 14% of total change) while the growth of more highly educated women tended to increase it (0.008, 16%) though not to the point of offsetting the general trend downwards.

The pattern of change among the baby bust cohorts (born 1950–1969) is radically different because the shift towards higher levels of childlessness was dominated by composition effects (0.056, 92% of the total change in the pooled model). Specifically, the growth of childlessness occurred mostly (0.048, 79% of the total change) as a result of changes in marital status with ever-higher proportions of never-married women (see the change in distributions in Table 1). In addition, the higher educational attainment among Spanish women from these cohorts, especially increasing numbers of women with higher education, also accounts for a considerable part of the growth in childlessness (0.012, 20% for all educational levels; 0.010, 17% for university graduates).

Rates effects among these cohorts have the same sign but are much less powerful (0.005, 8% of the total change) than compositional effects. The important point here is that the compositional effects for increasing childlessness of marital status outweighed the growing tendency to have children of never-married women (rate effect = − 0.003). The increase in childlessness was also the consequence of a non-negligible rate effect of ever-married women (represented in the decomposition by the constant term). In other words, without compositional changes in marital status among cohorts born after 1950, their childbearing behaviour would have tended to lower the overall rate of childlessness in Spain and possibly nullify the upward trend observed in the data. The case of education is somewhat different because its overall net effect is much smaller: due to the educational gradient in childlessness, the extraordinary growth in education among Spanish women born 1950–1969 tended to increase childlessness (an aggregate composition effect of 0.012 for all levels above primary) more than offsetting changes in childbearing behaviour that tended to reduce it (an aggregate rate effect of − 0.002 for the same educational levels).

## Discussion and Conclusions

This study provides new and accurate evidence regarding the dual trend in childlessness experienced by Spanish women born 1920–1969. Among women born 1920–1954 it declined substantially and then rose again among women born 1955–1969. Decreases in childlessness for cohorts born in the first half of twentieth century have appeared in many different nations and are considered an essential component of the baby boom (van Bavel and Reher 2013; Reher and Requena 2015a, b). The increases among more recent cohorts may also be a critical component of contemporary society, though further empirical studies are necessary before this statement can be accepted at face value. The changes implied in this dual trend can be interpreted as a transition from traditional to modern forms of childlessness. As a result of such a transition, the socio-demographic correlates of modern childlessness are very different from the old one, and recent empirical research has shown that in some developed societies the relationship between the classic components of childlessness (basically, nuptiality and education) may have begun to change (Pagnini and Rindfuss 1993; González and Jurado-Guerrero 2006; Hoem et al. 2006; Tanturri and Mencarini 2008; Beaujouan et al. 2015), though it is not easy to pinpoint just where that change is moving and how far it will go.

In Spain, traditional childlessness, as represented by the 1920–1924 cohort, can be largely understood in terms of (1) the structural imbalances in the marriage market caused by predominantly male overseas migration and excess male mortality (Muñoz-Pérez and Recaño-Valverde 2011) and (2) material deprivation (poverty, health problems, poor nutrition) chiefly associated to disadvantaged (less educated) social segments. Here it is worth remembering that, although the relationship between social status and childlessness is positive and strong, the majority of childless women born in 1920–1924 were concentrated among those with lower education and hence can be understood as the product of low socioeconomic status. In contrast, among the female population born in the 1960s most childlessness is accounted for by high levels of women never marrying and, due to the educational make-up of this female population, it affected preferentially women of medium and higher educational attainment; that is, those belonging to relatively higher social strata. All of these factors indicate that modern childlessness is not driven by the same social and demographic forces that operated in more traditional contexts and ultimately suggest that attitudes with regards to reproduction and childbearing were playing a more relevant role than in the past.

In other words, a large part of modern female childlessness no longer appears to be driven by a straightforward lack of men (related to skewed marriage markets due to migration or to war), health problems (including the incidence of miscarriages) or pure and simple destitution. There could also be another, paradigmatically new, form of male shortage affecting these female cohorts because of a marriage market squeeze suffered in recent decades by a growing number of highly educated Spanish women who appear to have been reluctant to marry down in educational terms (González 2003). In any case, for these more recent cohorts a new set of priorities come into play, at least insofar as childlessness does not seem to be an automatic outcome of traditional limitations to childbearing, but rather the result of an enhanced level of human (individuals or couples) agency. Thus the traditional structural constraints imposed by the marriage markets and individual and socioeconomic disadvantages that curbed the opportunities for having children weakened and made room to new types of behaviour where a higher degree of choice existed. In these new societal contexts, the cultural constraints on childlessness were relaxed allowing women to have competing goals (Sobotka and Testa 2008; Seiz 2013). In this sense, the women from these more recent cohorts are likely the harbingers in Spain of the type of new and more approving attitude towards voluntary childlessness that has been detected recently in contemporary European countries (Merz and Liefbroer 2012). At the very least, the opportunity costs of remaining childless must have changed in an important way.

The robustness of these results are limited by the fact there is no way to assess the role of cohabitation for childlessness because this variable is not present in census data. In order to assess the importance of this issue, we have undertaken an auxiliary analysis of the 2006 Fertility and Values Survey in which questions on the experience of cohabitation were included. This analysis made use of three cohorts (women born 1920–1934, 1935–1949 and 1950–1969), approximately reflecting those used in this paper. For the first two cohorts, the incidence of different forms of cohabitation among never-married women (2.1 and 2.0%) suggests that it was basically irrelevant for the purposes of our analysis. In the more recent cohort, the overall importance of cohabitation among never-married women was slightly higher (4.7%), but still relatively small. In a multivariable analysis of this last cohort, results showed that the prevalence of childlessness was far higher among women having cohabited than it was among married women, though slightly lower than it was for never-married women who never cohabited. On this point, it should be noted that the survey results also show that proportions of single women—independent of any previous experience of cohabitation—rose significantly among cohorts born after 1950, thus confirming the rise in singlehood found in the Census that accounts for the recent increase in childlessness.^16^ These results fully confirm the robustness of those found in this paper and vindicate the role of marriage for childlessness despite rising levels of non-marital fertility. They also suggest that as the incidence of cohabitation increases in the future, a trend currently unabated in Spanish society, cohabitation could emerge as an important buffer between levels of childlessness among women of different marital status.

In the final analysis, the dynamics of childlessness in Spain shares some relevant characteristics with other advanced societies. Similarities include the changing role played by marital status (Kiernan 1999; Castiglioni and Dalla Zuanna 2009; Portanti and Whitworth 2009) and educational differentials (Wood et al. 2014; Beaujouan et al. 2015). During the recent period of increasing childlessness, the proportions of married women have steadily decreased and the association between marriage and childbearing has weakened due to increasing non-marital fertility among baby bust cohorts (Reher 2015). At the same time, concurrently with the dramatic increase in female educational attainment, the strong and positive educational gradient of childlessness observed among the earlier Spanish cohorts lessened because of the growing convergence between women of lower and medium levels of education. In line with other European countries, Spanish college graduates of the more recent cohorts studied have also shown a propensity to remain childless closer to levels shown by women of lower and medium educational attainment, as seen in some other European countries (Wood et al. 2014; Beaujouan et al. 2015).

A careful look at the results of a recent study of childlessness in the USA (Hayford 2013) in comparison with those found for Spain may prove to be instructive for a number of these points. These two recent studies of childlessness share a common thread in which the effects of both nuptiality and education on childlessness are examined specifically and jointly. Both of them have led to similar conclusions: there was a transformation in childlessness from earlier higher levels in which marriage cycles were the key factor, to a more contemporary one where marriage continues to be important though there are important changes in the way it affects childlessness. In both countries, the weight of changing marital status with the growth of unmarried women was decisive in accounting for the growth of childlessness. Furthermore, in both cases the traditional positive association between educational attainment and childlessness has been shown to change substantially, giving way to a more nuanced relationship that is quite different from the one holding among earlier cohorts. Undoubtedly, if these shared patterns could be observed in, and generalized to, others countries, we would be in a much better position to understand the dynamics of the modern cycle of childlessness.

The scope of models used in this study is constrained by two basic facts: the availability of data and the meaning of change over time. In this sense, they provide only a very limited view of a very large process of social change. During the period studied, a whole host of factors came into play in Spain that are central to any causal explanation of the process at work. These include the health of potential mothers, changes in marriage patterns including a significant increase in the age of entry into motherhood, the incidence and acceptability of having children out of wedlock, the prevailing attitudes towards marriage and celibacy, the availability of contraception, the significance of having a family, the role of women in society and their participation in the labour market, changes in existing cultural priorities and many more. All of these were present and all of them changed substantially over time. Yet none of them are included in our database or are available at an individual level. Without them, the models specified here offer little more than a hint at what was really happening. Secondly, the meaning of some of the variables that do exist change completely over time, in part as the consequence of the processes of social and cultural change mentioned above. Highly educated women were the true odd balls of the 1920s birth cohorts (spinsters, teachers, librarians, etc.), yet become the standard bearers for the rest of society for women born in the 1960s. Among these women non-marital fertility skyrocketed (Billari and Kohler 2004), the age of first birth was postponed (Martín-García and Baizán 2006), and cohabitation became widespread and was amply accepted (Dominguez-Folgueras and Castro-Martin 2013). Marriage as a preordained context for reproduction was an unquestioned value for the early cohorts, but only a very relative one for the later cohorts whose reproductive life took place at a time when both divorce and abortion were legal and socially acceptable. The limitations of these models should not prevent us from using them to visualize a much grander period of change that swept much of traditional Spain aside in favour of the brave new world of modern, individualist society.

In spite of the similarities in childlessness that Spain shares with other developed countries, interesting disparities also emerge. (1) The key trend changes in Spain appear to lag behind those in the USA by approximately 15 birth cohorts, and about 7–8 years behind the main changes in the UK. (2) For much of the century, especially after cohorts born in the 1920s, childlessness in Spain appears to be substantially higher than in the USA or the UK, and where this is not the case it is mostly the result of the lagged pattern of change in Spain.^17^ (3) Looking towards the future and related to these points, the period of high childlessness appears to have come to an end in the USA and the UK, though this is not the case in Spain where levels continue to increase, at least according to the 2011 census.

The delay in the Spanish cycle can be explained by the fact that the demographic transition came later than in the USA, as did the baby boom, the second demographic transition and a revolution in education that was already in full force in the USA among women born early in the twentieth century but in Spain did not affect the growth in secondary and higher education before women born during the 1940s and especially the 1950s. Spain was a latecomer to the banquet of modernization and its implications. It also shows that childlessness may be a useful indicator for measuring the overall pace of social change. Beyond this, however, the persistently higher levels of childlessness in Spain (with the sole exception of levels among those born at the beginning of the century) is an intriguing issue that may have no simple answer. For some reason, remaining childless has always been more common and may have been perceived as more acceptable in Spain than it ever was in the US, at least among cohorts born during the twentieth century.

The debate regarding the near future of childlessness is open (Tanturri et al. 2015). There is some indication that the current cycle of increasing childlessness has come to an end in the USA and in Great Britain coinciding with an end of cohort fertility decline and with what some authors have termed the completion of the second part of the ‘gender revolution’ in much of American society (Goldscheider et al. 2015). Judging from Spanish data, however, at present there is no indication of an end to increasing levels of childlessness. Projections of future trends under different assumptions all suggest that levels will continue to increase to somewhere between 23 and 27% in the near future. In Spain the gender revolution is still far from complete and levels of education will continue to increase among cohorts born during the 1970s. The trend towards increasing childlessness is likely to come to an end as these social and cultural transformations are completed. Judging by the experience of the US, this will happen among cohorts born during the latter half of the 1970s and will be at levels of childlessness that significantly higher than in the USA. Whether or not this will lead to a lasting rebound in cohort fertility is a matter of speculation but should not be discarded.

## Footnotes

1 Recent projections suggest that rates of childlessness may increase among cohorts born in the second half of 1970s (Esteve et al. 2016). Considering that levels of childlessness among women born in 1970s are substantially higher than they ever were in the past, this would not be surprising.

2 Evidence against this reversal in the impact of education on childlessness has also been found in the cases of Austria (Neyer and Hoem 2008) and Britain (Kneale and Joshi 2008; Portanti and Whitworth 2009).

3 Detailed methodological information on this source is available at the web site of Spanish Instituto Nacional de Estadística: http://www.ine.es/prodyser/micro_censopv.htm.

4 Tracking first order births for the 1965–1969 cohorts during the period 2011–2015 in Spanish vital statistics (Movimiento Natural de la Población) shows that the 2011 Census data overestimate overall levels of childlessness by about 0.5%.

5 Murphy (2009) and Ní Bhrolcháin et al. (2011) deal with the issue of non-response and childlessness in the case of surveys.

6 Comparing the estimated percentages of childlessness for the thirty single year cohorts born 1920–1951 present on both the 1991 and 2011 censuses, the maximum difference in childlessness between them is 2.89% and the average difference is 1.21%. These results are satisfactory and suggest that data quality on the 2011 census is acceptable.

7 While there is no way to estimate accurately the differences in children ever born among both types of women, the 1981 Spanish Census makes some approximation possible. The weight of never-married women is much higher among institutionalized populations than among people residing in households. For example, for women 60–64 on the 1981 census, 12.2% of those living in households were never-married as opposed to 85% never-married in institutions. For women 80–84 present on the same census these percentages are 12.4 and 50.8%. On the same census and for the same age groups, the overall weight of the institutionalized population is 1.2% (women 60–64) and 4.7% (women 80–84). Considering all women above 50, these data suggest that the overall percentage of never-married women (12.8%), at least on the 1981 census, is underestimated by 1% when estimates are based only on women living in households (11.7%).

8 Lower education category includes women with primary education or less; medium education corresponds to lower and upper secondary education; and higher refers to university education. In order to strengthen the robustness of our empirical analysis, all the models have also been run with education coded into five categories (less than primary, primary, low secondary, upper secondary, and university). The results of both codifications are similar. In the interest of clarity and parsimony only the three-category model results are presented here.

9 This variable is based on the current administrative division of the country into autonomous regions according to the following groupings: North (= Galicia, Asturias, Cantabria, País Vasco), North Centre (= Aragon, Castilla–León, Navarra, Rioja), Centre (= Castilla La Mancha, Madrid), East (= Cataluña, Baleares, Valencia, Murcia), and South (= Andalucía, Extremadura, Canarias, Ceuta, Melilla).

10 The variable ‘Rural–urban divide’ is based on the size of municipality: rural (< 2000 inhabitants), small cities (2000–100,000 inhabitants), intermediate (100,001–500,000 inhabitants), and big cities (> 500,000 inhabitants).

11 Other estimations are available upon request from the authors.

12 Toulemon (1996) estimated that 8% of women trying to get pregnant for the first time when they are aged 25 do not succeed without some form of therapy. According to data from the 1991 Socio-demographic Survey, Spanish cohorts born in 1920s had their first child on average at the age of 26.8 (Requena 1997).

13 In order to verify whether coefficients differ significantly across cohorts, a pooled model with interactions for cohort and education has been specified. The results, not shown here (but available from the authors upon request), are clear: as implicit in Model 4 of Table 2, except for the first two cohorts, the interaction coefficients are statistically significant, showing real differences among cohorts.

14 Coefficients for the rural–urban divide and regions do not exhibit a single pattern across the cohorts. The models shown here do not capture the variations over time of these variables because of shifting patterns of internal migration that were very relevant in the 1960s: women living in urban settings in 2011 could well have been born and spent much of their fertile period in rural areas; and women living in one region at the census date could have been residing in another region during their reproductive years. There are also important changes over time in the effects of migratory status: while being native is significantly associated with lower levels of childlessness among the women born 1925–1959, coefficients for native women born in 1960’s are positive and are the result of higher levels of fertility among immigrant women arriving in Spain at the outset of the 21st century (cf. González-Ferrer et al. 2017). This interpretation of fertility differentials between native and immigrant women across cohorts should be further qualified in the sense that census data do not offer any information about the date of birth of children, so we do not know when and where immigrant women had their children (before or after migrating).

15 This same pattern of increasing disassociation of celibacy and childlessness has been also observed in the USA for cohorts born 1931–1968, with the uncoupling of marriage and reproduction working both ways (Hayford 2013).

16 The results of this analysis can be obtained from the authors upon request.

17 This becomes very clear if both time series are centered on the specific timing of the trend changes in each context.

## References

[CR1] Abma JC, Martinez GM (2006). Childlessness among older women in the United States: Trends and profiles. Journal of Marriage and Family.

[CR2] Beaujouan, E., Brzozowska, Z., & Zeman, K. (2015). *Childlessness trends in twentieth-century Europe: Limited link to growing educational attainment*. Vienna Institute of Demography, Working Papers, 6.

[CR3] Billari F, Kohler H-P (2004). Patterns of low and lowest-low fertility in Europe. Population Studies.

[CR4] Blinder AS (1973). Wage discrimination: Reduced form and structural variables. Journal of Human Resources.

[CR5] Blossfeld H-P, De Rose A (1992). Educational expansion and changes in entry into marriage and motherhood. The experience of Italian women. Genus.

[CR6] Blossfeld H-P, Jaenichen U (1992). Educational expansion and changes in women’s entry into marriage and motherhood in the Federal Republic of Germany. Journal of Marriage and the Family.

[CR7] Caldwell J (1980). Mass education as a determinant of the timing of fertility decline. Population and Development Review.

[CR8] Castiglioni M, Dalla Zuanna G (2009). Marital and reproductive behavior in Italy after 1995: Bridging the gap with Western Europe?. European Journal of Population.

[CR9] Coale A, Watkins SC (1986). The decline of fertility in Europe.

[CR10] Daguet F (2000). L’évolution de la fécondité des générations nées de 1917 à 1949: analyse par rang de naissance et niveau de diplôme, estimation d’aprés les enquêtes Famille de l’Insee. Population.

[CR11] David PA, Sanderson W (1990). Cohort parity analysis and fertility transition dynamics: Reconstructing historical trends in fertility control from a single census. Population Studies.

[CR12] Devolder D, Torres C (2015). Fecundidad: factores de la baja fecundidad en España. España 2015. Situación social [Spain 2015. Social Situation].

[CR01] Devolder D, Nicolau R, Panareda E (2006). La fecundidad de las generaciones españolas nacidas en la primera mitad del siglo XX. Un estudio a nivel provincial. Revista de Demografía Histórica.

[CR13] Dominguez-Folgueras M, Castro-Martin T (2013). Cohabitation in Spain: No longer a marginal path to family formation. Journal of Marriage and Family.

[CR14] El-Badry MA (1961). Failure to make entries of zero: Errors in recording childless in population censuses. Journal of the American Statistical Association.

[CR15] Esteve A, Devolder D, Domingo A (2016). Childlessness in Spain: Tick tock, tick tock, tick tock!. Perspectives Demogràfiques.

[CR16] Fairlie RW (1999). The absence of the African–American owned business: An analysis of the dynamics of self-employment. Journal of Labor Economics.

[CR17] Fairlie RW (2005). An extension of the Blinder–Oaxaca decomposition technique to logit and probit models. Journal of Economic and Social Measurement.

[CR18] Feeney, G. (1995). *The analysis of children ever born data for post-reproductive age women*. Notestein Seminar, Office of Population Research, Princeton University.

[CR19] Fernández Cordón JA, Olano A (1986). Análisis longitudinal de la fecundidad en España’. Tendencias demográficas y planificación económica [Demographic Trends and Economic Planning].

[CR20] Goldscheider F, Bernhardt E, Lappegård T (2015). The gender revolution: A framework for understanding changing family and demographic behavior. Population and Development Review.

[CR21] González MJ, Blossfeld H-P, Timm A (2003). Who marries whom in Spain?. Who marries whom? Educational systems as marriage markets in modern societies.

[CR22] González MJ, Jurado-Guerrero T (2006). Remaining childless in affluent economies: A comparison of France, West Germany, Italy and Spain, 1994–2001. European Journal of Population.

[CR23] González-Ferrer A, Castro-Martín T, Kraus EE, Eremenko T (2017). Childbearing patterns among immigrant women and their daughters in Spain: Over-adaptation or structural constraints?. Demographic Research.

[CR24] Hayford SR (2013). Marriage (still) matters: The contribution of demographic change to trends in childlessness in the United States. Demography.

[CR25] Hicks WW, Martínez-Aguado T (1987). Los determinantes de la fecundidad dentro del matrimonio en España. Revista Española de Investigaciones Sociológicas.

[CR26] Hirschman Ch (1994). Why fertility changes. Annual Review of Sociology.

[CR27] Hoem JM, Neyer G, Andersson G (2006). Education and childlessness: The relationship between educational field, educational level, and childlessness among Swedish women born in 1955–59. Demographic Research.

[CR28] Jann, B. (2006). FAIRLIE: Stata module to generate nonlinear decomposition of binary outcome differentials. Statistical Software Components S456727, Boston College Department of Economics. http://ideas.repec.org/c/boc/bocode/s456727.html. Accessed May 2007.

[CR29] Kiernan K (1999). Childbearing outside marriage in Western Europe. Population Trends.

[CR30] Kneale D, Joshi H (2008). Postponement and childlessness: Evidence from two British cohorts. Demographic Research.

[CR31] Martín-García T, Baizán P (2006). The impact of the type of education and of educational enrolment on first births. European Sociological Review.

[CR32] Masson L (2013). Avez-vous eu des enfants ? Si oui, combien?. France, portrait social—edition 2013.

[CR33] Merz EM, Liefbroer AC (2012). The attitude toward voluntary childlessness in Europe: Cultural and institutional explanations. Journal of Marriage and Family.

[CR34] Monstad K, Propper C, Salvanes K (2008). Education and fertility: Evidence from a natural experiment. Scandinavian Journal of Economics.

[CR35] Morgan SP (1991). Late nineteenth and early twentieth-century childlessness. American Journal of Sociology.

[CR36] Muñoz-Pérez F (1995). Procreación y matrimonio en España (1970–1990). Revista Internacional de Sociología.

[CR37] Muñoz-Pérez F, Recaño-Valverde J (2011). A century of nuptiality in Spain, 1900–2007. European Journal of Population.

[CR38] Murphy M (2009). Where have all the children gone? Women’s reports of more childlessness at older ages than when they were younger in a large-scale continuous household survey in Britain. Population Studies.

[CR39] Neyer G, Hoem JM, Surkyn J, van Bavel J, Deboosere P (2008). Education and permanent childlessness: Austria versus Sweden: A research note. Demographic challenges for the 21st century: A state of the art in demography.

[CR40] Ní Bhrolcháin MN, Beaujouan E, Murphy M (2011). Sources of error in reported childlessness in a continuous British household survey. Population Studies.

[CR41] Nicolau R, Devolder D, Panareda E (2010). La modernización de los comportamientos de fecundidad en España durante el siglo XX. Un estudio a nivel provincial para las generaciones nacidas en la primera mitad del siglo XX. Papers.

[CR42] Oaxaca RL (1973). Male–female wage differentials in urban labor markets. International Economic Review.

[CR43] Pagnini D, Rindfuss RR (1993). The divorce of marriage and childbearing: Changing attitudes and behavior in the United States. Population and Development Review.

[CR44] Portanti M, Whitworth S (2009). A comparison of the characteristics of childless women and mothers in the ONS longitudinal study. Population Trends.

[CR45] Reher D (2015). Baby booms, busts, and population ageing in the developed world. Population Studies.

[CR46] Reher D, Iriso PL (1989). Marital fertility and its determinants in Spain, 1887–1920. Population Studies.

[CR47] Reher D, Requena M (2015). The mid-twentieth century fertility boom from a global perspective. The History of the Family.

[CR48] Reher D, Requena M (2015). Was there a mid-twentieth century fertility boom in Latin America?. Revista de Historia Económica/Journal of Iberian and Latin American Economic History.

[CR49] Requena M (1997). Sobre el calendario reproductivo de las mujeres españolas. Revista Española de Investigaciones Sociológicas.

[CR50] Requena M, Salazar L (2006). El papel de la educación en la transición demográfica de las mujeres madrileñas. Revista Internacional de Sociología.

[CR51] Requena M, Salazar L (2014). Education, marriage and fertility: The Spanish case. Journal of Family History.

[CR52] Rindfuss RR, Bumpass L, St. John C (1980). Education and fertility: Implications for the roles women occupy. American Sociological Review.

[CR53] Rowland DT (2007). Historical trends in childlessness. Journal of Family Issues.

[CR54] Seiz M (2013). Voluntary childlessness in southern Europe: The case of Spain. Population Review.

[CR55] Sobotka, T. (2016). *Childlessness in Europe: Reconstructing long*-*term trends among women born in 1900*–*1972*. Paper presented at PPA 2016 annual meeting, Washington, DC, March 31 to April 2 2016.

[CR56] Sobotka T, Testa MR, Höhn Ch, Avramov D, Kotowska I (2008). Attitudes and intentions toward childlessness in Europe. People, population change and policies lessons from the population policy acceptance study.

[CR57] Stearns E, Moller S, Blau J, Potochnick S (2007). Staying back and dropping out: The relationship between grade retention and school dropout. Sociology of Education.

[CR58] Tanturri ML, Mencarini L (2008). Childless or childfree? Paths of voluntary childlessness in Europe. Population and Development Review.

[CR59] Tanturri, M. L., Mills, M., Rotkirch, A., Sobotka, T., Takács, J., Miettinen, A., Faludi, C., Kantsa, V., & Nasiri, D. (2015). *Childlessness in Europe*. Families and Societies, Working Paper Series, 32.

[CR60] Toulemon L (1996). Very few couples remain voluntarily childless. Population: An English Selection.

[CR61] van Bavel J, Reher D (2013). The baby boom and its causes: What we know and what we need to know. Population and Development Review.

[CR62] Van de Walle E, Glass DV, Revelle R (1972). Marriage and marital fertility. Population and social change.

[CR63] Van Hook J, Brown SL, Kwenda MN (2004). A decomposition of trends in poverty among children of immigrants. Demography.

[CR64] Wood J, Neels K, Kil T (2014). The educational gradient of childlessness and cohort parity progression in 14 low fertility countries. Demographic Research.

[CR65] Wrigley EA (1987). People, cities and wealth. The transformation of traditional society.

[CR66] Wunsch G, Ruzicka LT (1982). Effect of changes in nuptiality on natality in western Europe. Nuptiality and fertility.

